# Cudraflavone C Induces Apoptosis of A375.S2 Melanoma Cells through Mitochondrial ROS Production and MAPK Activation

**DOI:** 10.3390/ijms18071508

**Published:** 2017-07-13

**Authors:** Chiang-Wen Lee, Feng-Lin Yen, Horng-Huey Ko, Shu-Yu Li, Yao-Chang Chiang, Ming-Hsueh Lee, Ming-Horng Tsai, Lee-Fen Hsu

**Affiliations:** 1 Department of Nursing, Division of Basic Medical Sciences, Chang Gung University of Science and Technology, Chia-Yi 61363, Taiwan; cwlee@mail.cgust.edu.tw (C.-W.L.); yaochang.chiang@gmail.com (Y.-C.C.); 2 Chronic Diseases and Health Promotion Research Center, Chang Gung University of Science and Technology, Chia-Yi 61363, Taiwan; 3 Research Center for Industry of Human Ecology, Chang Gung University of Science and Technology, Taoyuan 33303, Taiwan; 4 Department of Fragrance and Cosmetic Science, College of Pharmacy, Kaohsiung Medical University, Kaohsiung 80708, Taiwan; flyen@cc.kmu.edu.tw (F.-L.Y.); hhko@kmu.edu.tw (H.-H.K.); 5 Institute of Biomedical Sciences, National Sun Yat-Sen University, Kaohsiung 80424, Taiwan; 6 Department of Pharmacy, College of Pharmacy & Health Care, Tajen University, Pingtung 90741, Taiwan; syli@mail.tajen.edu.tw; 7 Center for Drug Abuse and Addiction, China Medical University Hospital, China Medical University, Taichung 40447, Taiwan; 8 Division of Neurosurgery, Department of Surgery, Chang Gung Memorial Hospital, Chia-Yi61363, Taiwan; ma2072@gmail.com; 9 Department of Pediatrics, Division of Neonatology and Pediatric Hematology/Oncology, Chang Gung Memorial Hospital, Yunlin 63862, Taiwan; mingmin.tw@yahoo.com.tw; 10 Department of Respiratory Care, Chang Gung University of Science and Technology, Chia-Yi Campus, Chia-Yi 61363, Taiwan

**Keywords:** cudraflavone C, mitochondria, melanoma cells, MAPKs, pro-oxidation, apoptosis

## Abstract

Melanoma is the most malignant form of skin cancer and is associated with a very poor prognosis. The aim of this study was to evaluate the apoptotic effects of cudraflavone C on A375.S2 melanoma cells and to determine the underlying mechanisms involved in apoptosis. Cell viability was determined using the MTT and real-time cytotoxicity assays. Flow cytometric evaluation of apoptosis was performed after staining the cells with Annexin V-FITC and propidium iodide. The mitochondrial membrane potential was evaluated using the JC-1 assay. Cellular ROS production was measured using the CellROX assay, while mitochondrial ROS production was evaluated using the MitoSOX assay. It was observed that cudraflavone C inhibited growth in A375.S2 melanoma cells, and promoted apoptosis via the mitochondrial pathway mediated by increased mitochondrial ROS production. In addition, cudraflavone C induced phosphorylation of MAPKs (p38, ERK, and JNK) and up-regulated the expression of apoptotic proteins (Puma, Bax, Bad, Bid, Apaf-1, cytochrome C, caspase-9, and caspase-3/7) in A375.S2 cells. Pretreatment of A375.S2 cells with MitoTEMPOL (a mitochondria-targeted antioxidant) attenuated the phosphorylation of MAPKs, expression of apoptotic proteins, and the overall progression of apoptosis. In summary, cudraflavone C induced apoptosis in A375.S2 melanoma cells by increasing mitochondrial ROS production; thus, activating p38, ERK, and JNK; and increasing the expression of apoptotic proteins. Therefore, cudraflavone C may be regarded as a potential form of treatment for malignant melanoma.

## 1. Introduction

Melanoma is a common form of cancer, particularly among Caucasians. It is also the most malignant form of skin cancer, and is associated with a very poor prognosis. Currently, treatment modalities for malignant melanoma include surgical excision, chemotherapy, radiotherapy, and immunotherapy. However, these forms of treatment are often associated with severe adverse effects, and chances of long-term survival remain poor even after treatment [1,2]. Therefore, investigating new approaches for treating malignant melanoma is of paramount clinical importance.

**Figure 1:**
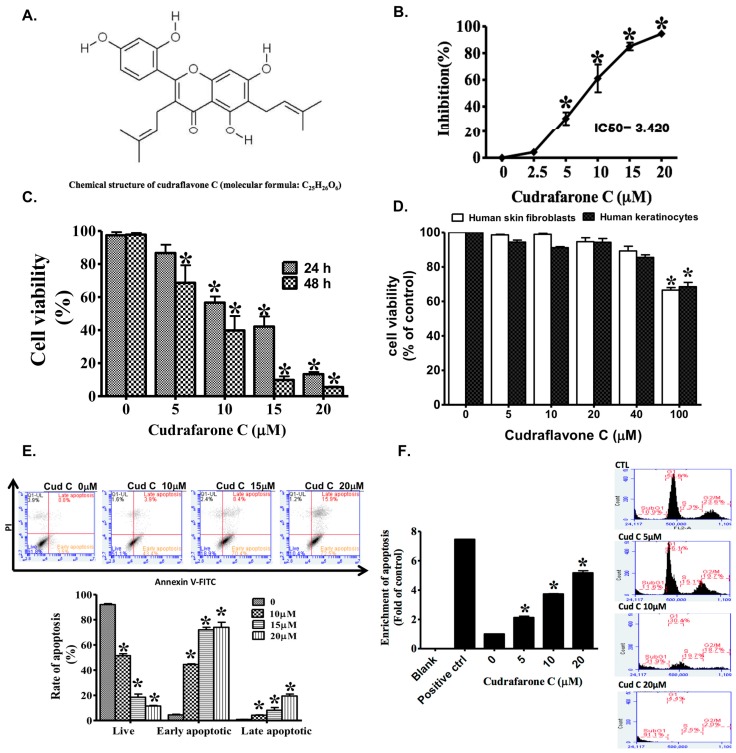
(**A**) Chemical structure of cudraflavone C; (**B**) Inhibition of A375.S2 cell proliferation by cudraflavone C, as determined by the SRB assay at 24 h; (**C**) Effects of cudraflavone C on cell viability in A375.S2 cells, as determined by the MTT assay at 24 and 48 h; (**D**) Effects of cudraflavone C on cell viability in human skin fibroblasts(open bars) and human keratinocytes (HaCaT cells) (shard bars), as determined by the MTT assay at 24 h; (**E**) Effects of cudraflavone C on cell apoptosis in A375.S2 cells, as determined by flow cytometry following AnnexinV-FITC and propidium iodide staining at 24 h. Cells in the right lower quadrant are undergoing early apoptosis; (**F**) Effects of cudraflavone C on cell apoptosis (determined by DNA fragmentation assay, left panel) and sub-G1 cell cycle arrest (determined by flow cytometry following propidium iodide staining, right panel) in A375.S2 cells at 24 h. (**B**–**F**) Results are shown as mean ± SEM of three independent experiments. * *p* < 0.05 compared to the control group.

Cudraflavone C (Figure 1A) is a naturally occurring flavonol that is derived from the roots of *Artocarpusxanthocarpus*. *Artocarpus* Sp. have been previously demonstrated to possess inhibitory activities against tyrosinase [3], pancreatic lipase [4], and the herpes simplex virus [5]. Additionally, studies have also demonstrated that *Artocarpus* Sp. possesses anticancer properties against melanoma [6], hepatocellular carcinoma, gastric carcinoma [7], and colorectal carcinoma [8]. However, the mechanisms underlying the anti-melanoma properties of cudraflavone C have not been investigated. Reactive oxygen species (ROS) play a dual role in biological systems [9,10,11]. Firstly, under physiological conditions, the generation of ROS plays important roles in phagocytosis, cell signaling, and homeostasis; however, these reactive species are subsequently eliminated by the scavenging system in normal cells [12]. Secondly, under conditions of oxidative stress, a higher accumulation of ROS oxidizes the cellular lipids, proteins, and DNA; thereby leading to the aggravation of many diseases (including cancer, cardiovascular diseases, and neurodegenerative disorders) and the promotion of aging and inflammation [13,14,15]. Previous studies have revealed that some anticancer drugs reported in traditional Chinese herbal medicine, such as paclitaxel [16], resveratrol [17], and curcumin [18], increased the production of ROS to suppress the growth of cancer cells by mediating the activation of mitogen-activated protein kinases (MAPKs) and the expression of apoptotic proteins. In this study, we evaluated the effects of cudraflavone C treatment on the proliferation and apoptosis of A375.S2 melanoma cells. Furthermore, we also determined the underlying mechanisms involved in these processes, including the production of ROS and signaling via the MAPK pathway.

## 2. Results

### 2.1. Cudraflavone C Inhibits Proliferation of A375.S2 Melanoma Cells

Using the SRB assay, it was shown that treatment of A375.S2 melanoma cells with cudraflavone C (2.5–20 µM) for 24 h inhibited cell proliferation in a concentration-dependent manner (Figure 1B) with an IC_50_ value of 3.420 µM. Furthermore, the results of the MTT assay demonstrated that treatment of A375.S2 cells with cudraflavone C for 24 or 48 h decreased cell viability in a concentration-dependent manner (Figure 1C). On the other hand, treatment of the human skin fibroblasts and HaCaT cells with cudraflavone C for 24 h did not significantly inhibit cell viability (as determined using the MTT assay) up to a concentration of 100 µM (Figure 1D). 

### 2.2. Cudraflavone C Promotes Apoptosis and Cell Cycle Arrest in A375.S2 Melanoma Cells

Apoptosis in A375.S2 cells was measure dusing flow cytometry after staining them with AnnexinV-FITC and propidium iodide. As shown in Figure 1E, cudraflavone C (10, 15, and 20 µM) promoted apoptosis in A375.S2 cells in a concentration-dependent manner. The percentage of cells undergoing early apoptosis (Figure 1E, right lower quadrant) after cudraflavone C treatment for 24 h were 3.5% (0 µM), 43.4% (10 µM), 70.4% (15 µM), and 72.5% (20 µM). Moreover, treatment of A375.S2 cells with cudraflavone C (5, 10, and 20 µM) for 24 h led to a concentration-dependent increase in DNA fragmentation (Figure 1F, left panel). In addition, treatment of A375.S2 cells with cudraflavone C resulted in cell cycle arrest with an increased percentage of cells being arrested in the sub-G1 phase (Figure 1F, right panel). The percentage of cells observed in the sub-G1 phase after cudraflavone C treatment for 24 h were 10.9% (0 µM), 11.9% (5 µM), 31.6% (10 µM), and 91.1% (20 µM).

### 2.3. Cudraflavone C Promotes Apoptosis in A375.S2 Cells via the Mitochondrial Pathway

**Figure 2:**
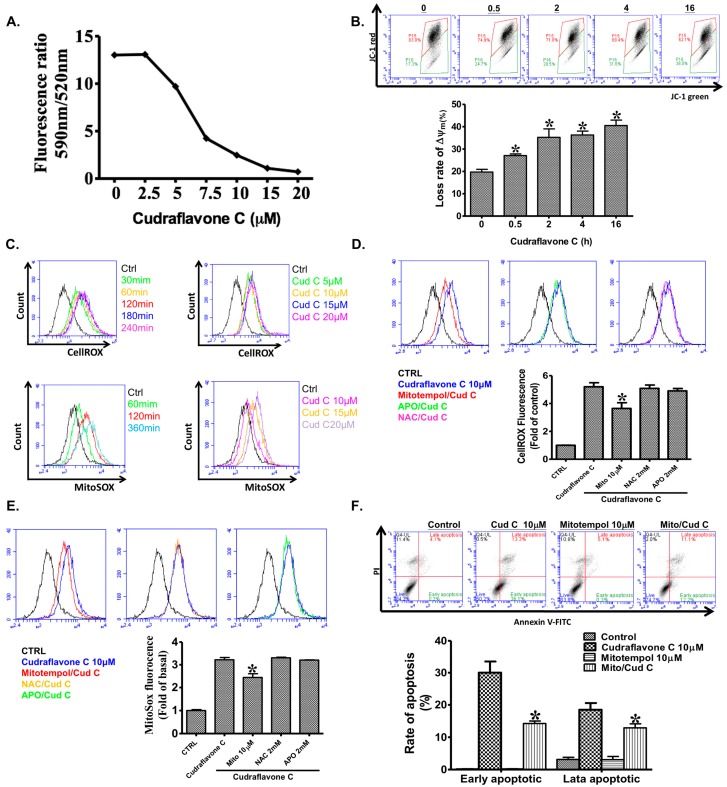
(**A**) Effect of cudraflavone C on mitochondrial membrane potential in A375.S2 cells, as determined by the JC-1 assay at 24; (**B**) A375.S2 cells were labeled with JC-1 (10 μg/mL) and then stimulated with cudraflavone C for the indicated times (6, 12 and 24 h). The ΔΨm results are representative of three independent experiments; (**C**) Effect of cudraflavone C on cellular ROS production (determined by flow cytometry after staining with CellROX reagent) in A375.S2 cells as a function of time (upper left panel) and concentration (upper right panel). Effect of cudraflavone C on mitochondrial ROS production (determined by flow cytometry after staining with MitoSOX Red indicator) in A375.S2 cells as a function of time (lower left panel) and concentration (lower right panel); (**D**) A375.S2 cells were pretreated for 1 h with mitochondria-targeted antioxidant (MitoTEMPOL), antioxidant (NAC), or NADPH oxidase inhibitor (APO) and then treated with cudraflavone C for 240 min, and cellular ROS production was determined by flow cytometry (after staining with CellROX reagent); (**E**) A375.S2 cells were pretreated for 1 h with MitoTEMPOL, NAC or APO and then treated with cudraflavone C for 360 min, and mitochondrial ROS production was determined by flow cytometry (after staining with MitoSOXRed indicator); (**F**) Effect of MitoTEMPOL (mitochondria-targeted antioxidant) on cudraflavone C-induced A375.S2 cell apoptosis (determined by flow cytometry following Annexin-V and propidium iodide staining). Cells in the right lower quadrant are undergoing early apoptosis; (**A**–**F**) Results are shown as mean ± SEM of three independent experiments. * *p* < 0.05 compared to the control group.

As shown in Figure 2A,B, treatment of A375.S2 cells with cudraflavone C (2.5–20 µM) for 24 h resulted in a decrease in the mitochondrial membrane potential in a concentration- and time-dependent manner, as determined by the JC-1 assay. This result indicated that cudraflavone C promoted apoptosis in the A375.S2 cells via the mitochondrial pathway.

### 2.4. Cudraflavone C Induces Mitochondrial ROS Production in A375.S2 Cells 

Cellular production of ROS was measured using the CellROX assay. As shown in Figure 2C, treatment of A375.S2 cells with cudraflavone C (10 µM) induced cellular ROS production in a time-dependent manner (Figure 2C, left upper panel). Moreover, treatment of the A375.S2 cells with different concentrations of cudraflavone C (5, 10, 15, and 20 µM) for 4 h resulted in increased cellular ROS production (Figure 2C, right upper panel). In addition, mitochondrial ROS production was determined using the MitoSOX assay. It was observed that treatment of the A375.S2 cells with cudraflavone C (10 µM) induced mitochondrial ROS production in a time-dependent manner (Figure 2B, left lower panel). Moreover, treatment of the A375.S2 cells with cudraflavone C (10, 15, and 20 µM) for 6h induced an increase in the mitochondrial ROS production (Figure 2B, right lower panel) in a concentration-dependent manner. Furthermore, inhibitors were used to confirm the source of ROS production in cells. As shown in Figure 2D, the increased production of cellular ROS (determined using the CellROX assay) induced by cudraflavone C (10 µM) was partially attenuated by MitoTEMPOL (mitochondria-targeted antioxidant, 10 µM), but not by the antioxidant (NAC, 2 mM) or NADPH oxidase inhibitor (APO, 2 mM). Similarly, as shown in Figure 2E, the increased production of mitochondrial ROS (assessed using the MitoSOX assay) induced by cudraflavone C (10 µM) was partially attenuated by MitoTEMPOL (10 µM), but not by NAC (2 mM) or APO (2 mM). These findings indicated that mitochondria, and not the NADPH oxidase, werethe main source of ROS production in A375.S2 cells treated with cudraflavone C.

### 2.5. Cudraflavone C Promotes Apoptosis in A375.S2 Cells via Mitochondrial ROS Generation

Apoptosis was measured using flow cytometry following AnnexinV-FITC and propidium iodide staining. As shown in Figure 2F, treatment of the A375.S2 cells with cudraflavone C (10 µM) for 24 h resulted in an increased percentage of cells undergoing early apoptosis (from 0.2% to 36.1%); however, this phenomenon was partially attenuated by pre-treating the cells with MitoTEMPOL (10 µM) for 1 h (cells undergoing early apoptosis, 12.2%). This indicated that cudraflavone C promoted apoptosis in the A375.S2 cells via mitochondrial ROS generation.

### 2.6. Cudraflavone C Induces Activation of the MAPK Pathway in A375.S2 Cells via Mitochondrial ROS Generation

**Figure 3:**
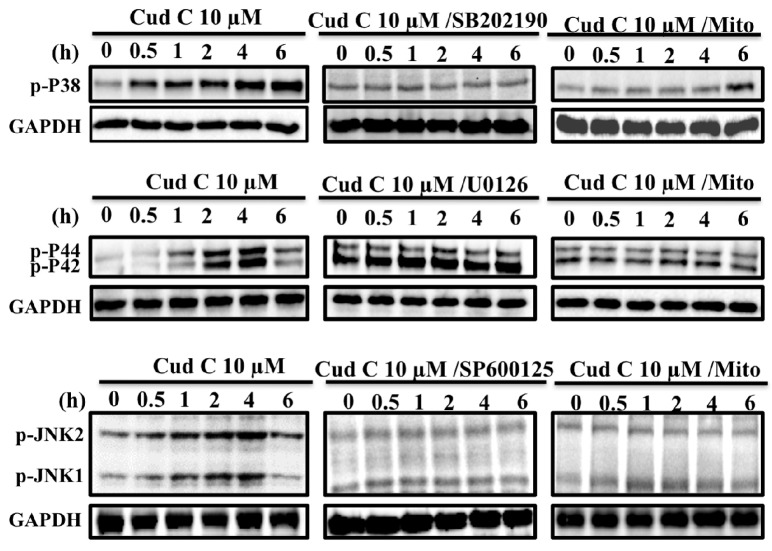
Effects of cudraflavone C on the phosphorylation status of p38, ERK (p44/p42) and JNK in A375.S2 cells over various time periods (0–6 h), as determined by Western blotting. In addition, cells were pretreated for 1 h with a p38 inhibitor (SB202190), MEK1/ERK inhibitor (U0126), JNK inhibitor (SP600125), and mitochondria-targeted antioxidant (MitoTEMPOL) and then treated with cudraflavone C, and the phosphorylation status of p38, ERK, and JNK was evaluatedover various time periods. GAPDH was used as a loading control. Blots were representative of three independent experiments.

The effects of cudraflavone C on MAPK activation in A375.S2 cells were investigated using Western blotting (Figure 3). It was observed that treatment of the A375.S2 cells with cudraflavone C (10 µM) induced phosphorylation of p38, ERK (p44/p42), and JNK in a time-dependent manner (from 0 to 6 h); however, these effects were suppressed by pre-treating the cells with their respective inhibitors (SB-202190, U-0126, SP600125) and by MitoTEMPOL (mitochondria-targeted antioxidant, 10 µM) for 1 h. This indicated that cudraflavone C induced activation of the MAPK pathway in A375.S2 melanoma cells via mitochondrial ROS generation.

### 2.7. Cudraflavone C Induces the Expression of Apoptotic Proteins in A375.S2 Cells via Mitochondrial ROS Generation

**Figure 4:**
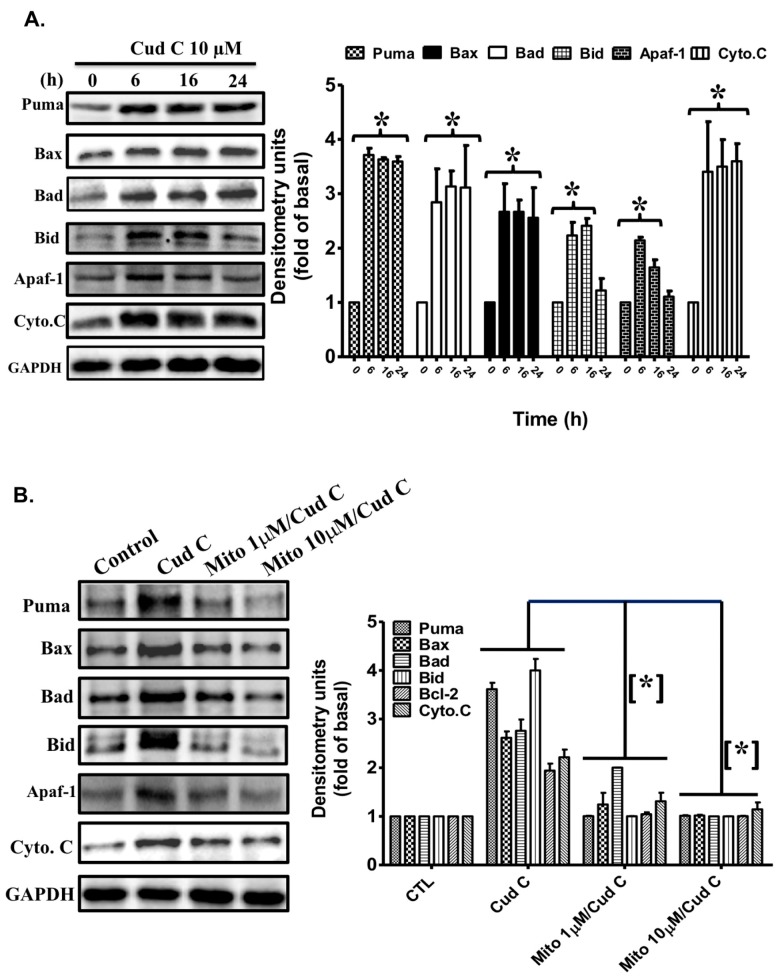
(**A**) Effects of cudraflavone C on the expression levels of apoptotic proteins Puma, Bax, Bad, Bid, Apaf-1 and cytochrome c in A375.S2 cells over various time periods (0–24 h), as determined by Western blotting; (**B**) Cells were pretreated with MitoTEMPOL (mitochondria-targeted antioxidant) for 1 h and then treated with cudraflavone C for 16 h, and the expression of apoptotic proteins was determined by Western blotting. GAPDH was used as a loading control. The intensity of the bands was quantified by densitometry, and data are expressed as mean ± SEM of three experiments. * *p* < 0.05 compared to the control group.

The effects of cudraflavone C on the expression of apoptotic proteins in A375.S2 cells were investigated using Western blotting (Figure 4A). It was observed that treatment of the A375.S2 cells with cudraflavone C (10 µM) promoted the expression of Puma, Bax, Bad, Bid, Apaf-1, and cytochrome c in a time-dependent manner (from 0 to 24 h). As shown in Figure 4B, the increased expression of Puma, Bax, Bad, Bid, Apaf-1, and cytochrome c induced by cudraflavone C (10 µM) in A375.S2 cells was attenuated by pre-treating the cells with MitoTEMPOL (1, 10 µM). This indicated that cudraflavone C promoted the expression of apoptotic proteins in A375.S2 cells which was mediated via mitochondrial ROS production.

### 2.8. Cudraflavone C Induces the Activation of Caspase-3, Caspase-7, and Caspase-9 in A375.S2 Cells

**Figure 5:**
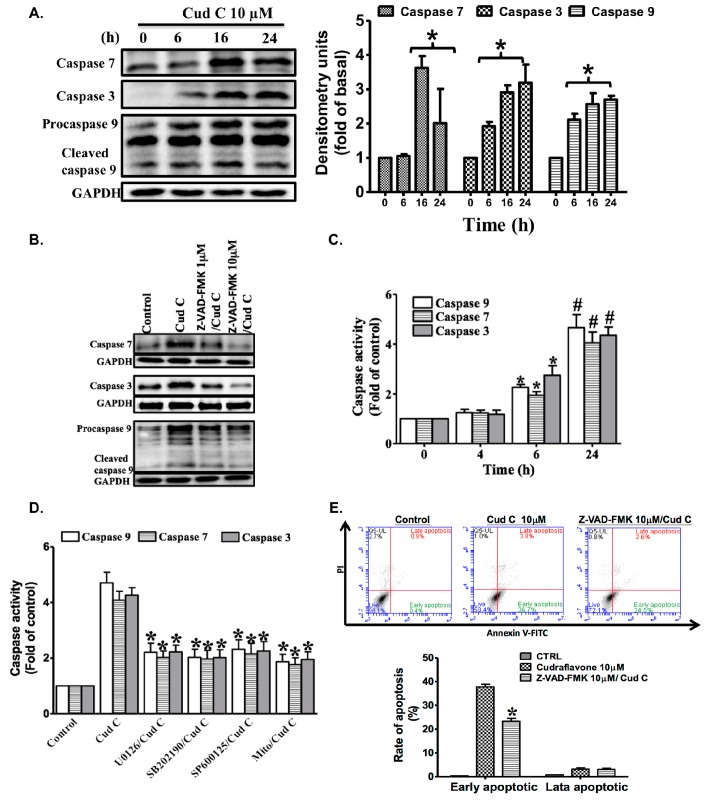
(**A**) Effects of cudraflavone C on the expression status of caspase-7, caspase-3, and caspase-9 in A375.S2 cells over various time periods (0–24 h), as determined by Western blotting; (**B**) In addition, cells were pretreated for 1 h with the caspase inhibitor Z-VAD-FMK and then treated with cudraflavone C for 16 h, and the levels of active caspase-7, -3, and -9 were evaluated by Western blotting; (**C,D**) Cells were treated with cudraflavone C for indicated times. The caspase activity was analyzed by using caspase-3, -7, and -9 colorimetric assay kits; (**E**) Confluent cells were pre-incubated with or without inhibitors of ERK1/2 (U0126), p38 (SB202190), JNK1/2 (SP600125) and MitoTEMPOL (mitochondria-targeted antioxidant). After incubation for 1 h, cells were treated with cudraflavone C (10 µM) for 24 h. The activities of caspases were analyzed by using caspase-3, -7, and -9 colorimetric assay kits. Results are representative of three independent experiments. * *p* < 0.05, ^#^ *p* < 0.01 compared to the control group.

The effects of cudraflavone C on the expression of active caspase-7, caspase-3, and caspase-9 in A375.S2 cells were investigated using Western blotting. As shown in Figure 5A, treatment of the A375.S2 cells with cudraflavone C (10 µM) increased the expression of caspase-7, caspase-3, and caspase-9 in a time-dependent manner (from 0 to 24 h). In addition, as shown in Figure 5B, the activation of caspase-7, caspase-3, and caspase-9 in A375.S2 cells was attenuated by pre-treating the cells with the caspase inhibitor Z-VAD-FMK (1, 10 µM). Moreover, in this study, we investigated the effects of cudraflavone C treatment on the activities of caspase-3, caspase-7, and caspase-9 in A375.S2 cells. The activities of these caspases were analyzed using the caspase-3, caspase-7, and caspase-9 colorimetric assay kits. As shown in Figure 5C, cudraflavone C (10 μM) markedly increased the activity of caspase-3, caspase-7, and caspase-9. Furthermore, confluent cells were pre-incubated with U-0126 (10 μM), SB-202190 (10 μM), SP600125 (10 μM), and MitoTEMPOL (10 µM) for 1 h, followed by stimulation with cudraflavone C (10 μM) for 24 h. As shown in Figure 5D, cudraflavone C treatment resulted in an increase in the activities of caspase-3, caspase-7, and caspase-9; these activities were attenuated by pre-treating the cells with U-0126, SB-202190, SP600125, and MitoTEMPOL. Finally, treatment with cudraflavone C (10 µM) for 24 h resulted in an increased percentage of cells undergoing early apoptosis (from 0.4% to 36.7%). This phenomenon was partially attenuated by pre-treating the cells with Z-VAD-FMK (10 µM) for 1 h (cells undergoing early apoptosis, 24.5%) (Figure 5E). These results suggested that cudraflavone C promoted apoptosis in A375.S2 cells via the mitochondrial and caspase pathways. 

## 3. Discussion

Skin cancers are the most common type of cancers in humans. Melanoma is the most malignant form of skin cancer and is associated with a high mortality rate [1]. Initially, melanoma may present itself clinically as pigmented or non-pigmented papules, plaques, or nodules anywhere on the skin, particularly in sun-exposed areas. However, the malignant cells rapidly metastasize to the regional lymph nodes and other visceral organs [2].Current treatment modalities for melanoma include surgery, radiotherapy, chemotherapy, immunotherapy, and the use of biological agents [19]. However, long-term survival chances for patients with melanoma remains poor even after aggressive treatment. For patients with Stage IV melanoma, the 5-year survival rate has been found to be only 9–28% [20]. Therefore, identification of novel compounds with potential therapeutic effects against melanoma is of immense clinical importance.

In recent years, various bioactive compounds derived from natural sources have been found to possess anticancer activities. In particular, flavonoids are a class of compounds that have been isolated from plants and are frequently associated with anticancer properties [21,22,23,24,25]. Different molecular mechanisms for the action of flavonoids on cancer cells have been proposed, including induction of apoptosis [26,27], generation of ROS [28,29], and signaling mediated via the MAPK [30] and Akt–mTOR pathways [31].

Cudraflavone C (Figure 1A) is a naturally occurring flavonol isolated from the roots of *Cudrania tricuspidata* and *Artocarpus* Sp. [32,33,34]. It has been shown to possess anti-tyrosinase [3,35], anti-melanin biosynthesis [36], anti-herpes simplex virus [5], antibacterial [37], and anti-pancreatic lipase [4,38] activities. In addition, the cytotoxic activities of cudraflavone C against colorectal cancer [8], hepatocellular and gastric carcinomas [7], and melanoma [6] have been demonstrated. However, the molecular mechanisms underlying the anti-melanoma property of cudraflavone C have not been investigated.

In this study, we observed that cudraflavone C inhibited the proliferation (IC_50_, 3.420 µM) and induced cytotoxicity in the A375.S2 melanoma cells. These activities were mediated via the induction of cell cycle arrest in the sub-G1 phase and promotion of apoptosis, as shown by the results of the DNA fragmentation assay and flow cytometric analysis. However, cudraflavone C did not demonstrate significant cytotoxicity towards normal human fibroblasts and keratinocytes (HaCaT cells) up to a concentration of 100 µM. These findings indicated that cudraflavone C may be used as a potent anticancer agent for the treatment of malignant melanoma. 

Apoptosis may occur following exposure of the cells to increased levels of oxidative stress. There are two main sources for ROS production in cells: NADPH oxidase, and mitochondria. We observed that cudraflavone C induced cellular and mitochondrial ROS production in A375.S2 cells, which could be attenuated by pre-treating the cells with MitoTEMPOL, but not with NAC (cellular antioxidant) or APO (NADPH oxidase inhibitor). In addition, the cudraflavone C-induced apoptosis in A375.S2 cells could be suppressed by pre-treating the cells with MitoTEMPOL. These findings indicated that the generation of ROS from mitochondria was a key event in mediating the apoptotic effects of cudraflavone C in the A375.S2 melanoma cells. In contrast, cellular NADPH oxidase did not appear to act as a prominent source of ROS following cudraflavone C treatment. Various signal transduction pathways may be activated upon induction of oxidative stress in cells. The MAPK signaling pathway is known to play a prominent role in cancer growth and metastasis. The three main types of MAPKs present in humans include ERK (p44/p42), JNK, and p38. ERK is mainly involved in cell proliferation, survival, migration, and invasion; while JNK and p38 are involved in cell apoptosis [39,40]. Our findings revealed that treatment of A375.S2 melanoma cells with cudraflavone C induced the activation and phosphorylation of MAPKs (p38, ERK, and JNK); however, these effects were suppressed by pre-treating the cells with specific MAPK inhibitors (SB-202190, U-0126, SP600125), or by a mitochondria-targeted antioxidant (MitoTEMPOL). Therefore, cudraflavone C-induced MAPK activation in A375.S2 melanoma cells may be mediated by mitochondrial ROS production. These findings were consistent with findings from previous studies which showed that mitochondrial ROS generation may induce MAPK activation leading to apoptosis [41].Apoptosis may be mediated by two distinct pathways: the death receptor pathway, and the mitochondrial pathway [42]. In the mitochondrial pathway, various types of cellular stress responses may cause translocation of pro-apoptotic proteins of the Bcl-2 family (Puma, Bax, Bad, Bid) from the cytosol to the mitochondria, resulting in increased mitochondrial permeability and the release of cytochrome c from the mitochondria [43,44]. Cytochrome c combines with Apaf-1 and procaspase-9 to form the apoptosome. This results in the sequential activation of caspase-9 and caspase-3/7 (effector caspases), and finally leads to apoptosis [45]. The results of the current study demonstrated that treatment of the A375.S2 melanoma cells with cudraflavone C induced increased expression of members of the pro-apoptotic Bcl-2 family (Puma, Bax, Bad, Bid), Apaf-1, and cytochrome c, which could be suppressed by pre-treating the cells with MitoTEMPOL. In addition, treatment of A375.S2 melanoma cells with cudraflavone C resulted in a decrease in the mitochondrial membrane potential (as determined by the JC-1 assay), and induced activation of caspase-9 and caspase-3/7. These findings indicated that cudraflavone C-induced cytotoxicity in melanoma cells was mediated by the generation of ROS from mitochondria and induction of apoptosis by the mitochondrial pathway. Our results were consistent with those of Sooet al. [8] who demonstrated that cudraflavone C induced apoptosis in colorectal cancer cells through the mitochondrial pathway. In conclusion, our findings demonstrated that cudraflavone C induced cytotoxicity in the A375.S2 melanoma cells through mitochondrial ROS production, MAPK (p38/ERK/JNK) activation, the expression of pro-apoptotic proteins of the Bcl-2 family (Puma, Bax, Bad, Bid), and the induction of apoptosis through the mitochondrial pathway. Therefore, cudraflavone C may well be considered as a potential form of treatment for malignant melanoma.

## 4. Materials and Methods

### 4.1. Materials

A human melanoma cell line (A375.S2) and a human epidermal keratinocyte cell line (HaCaT) were provided from one of co-author Feng-Lin Yen. Primary human fibroblasts were procured from the Food Industry Research and Development Institute (Hsinchu, Taiwan). Dulbecco’s Modified Eagle Medium/Nutrient Mixture F-12 (DMEM/F-12) was obtained from GIBCO (Grand Island, NY, USA) and supplemented with 10% fetal bovine serum (FBS) (Hazelton Research Products; Denver, PA, USA).Antibodies for Western blotting (phospho-p38, phospho-ERK, and phospho-JNK) were obtained from Cell Signaling Technology (Danvers, MA, USA). Puma, Bax, Bad, Bid, Apaf-1, cytochrome C, caspase-3, caspase-7, and caspase-9were obtained from Santa Cruz Biotechnology (Santa Cruz, CA, USA). The GAPDH antibody was obtained from Biogenesis (Bournemouth, UK). *N*-acetylcysteine (NAC), apocynin (APO), U-0126, SB-202190, and SP600125 were procured from Biomol (Plymouth Meeting, PA, USA). MitoSOX Red mitochondrial superoxide indicator was purchased from Molecular Probes (Eugene, OR, USA). Caspase-3, caspase-7, and caspase-9 colorimetric assay kits were obtained from R&D Systems (Minneapolis, MN, USA). Cudraflavone C was isolated from the methanolic extract of *A. xanthocarpus* as described previously in Reference [3], and its purity was determined (>97%) using high-performance liquid chromatography (HPLC) analysis.

### 4.2. Cell Culture

A375.S2, HaCaT, and human fibroblast cells were cultured in DMEM supplemented with 10% fetal bovine serum (FBS). Cells were maintained in a humidified incubator with 5% CO_2_ at 37 °C.

### 4.3. Cell Proliferation Assay

To assess cell proliferation, the sulforhodamine B (SRB) assay was performed. Briefly, A375.S2 cells were treated with varying concentrations of cudraflavone C for 24 h, and subsequently fixed in 10% trichloroacetic acid (TCA). Next, the cells were stained with SRB (Sigma-Aldrich, St. Louis, MO, USA) and washed with acetic acid (1%). Tris-base (10 mM) was added to dissolve the cell-bound SRB dye, and the absorbance was measured using a spectrometer at a wavelength of 515 nm.

### 4.4. Cell Viability Assay

Cell viability of A375.S2, HaCaT, and human fibroblast cells was determined using the MTT (3-(4,5-dimethylthiazol-2-yl)-2,5-diphenyltetrazolium bromide) assay (Sigma-Aldrich). Cells were plated onto 96-well plates and incubated overnight. The following morning, cells were treated with different concentrations of cudraflavone C for 24 or 48 h. Next, the MTT solution was added to the wells. After incubating for 1 h at 37 °C, the plates were analyzed using a plate reader at 550 nm.

### 4.5. Measuring Apoptosis Using Flow Cytometry

To measure cell apoptosis, the Annexin V-FITC/propidium iodide assay (Thermo Fisher Scientific) was performed. A375.S2 cells were treated with different concentrations of cudraflavone C (10, 15, and 20 µM) for 24 h, stained withRNAase, Annexin V-FITC and propidium iodide, andsubjected to flow cytometry. 

### 4.6. Measuring Apoptosis Using DNA Fragmentation Assay

Apoptosis was also measured by performing the DNA fragmentation assay using the Cell Death Detection ELISA kit (Roche Diagnostics, Basel, Switzerland). A375.S2 cells were treated with different concentrations of cudraflavone C (5, 10, and 20 µM) for 24 h, and the degree of DNA fragmentation was measured using an ELISA reader.

### 4.7. Cell Cycle Analysis

Following treatment with varying concentrations of cudraflavone C (5, 10, and 20 µM) for 24 h, the A375.S2 cells were stainedwith RNAase, propidium iodide and subjected to flow cytometry (Accuri C6 Flow Cytometer) (BD Biosciences, San Jose, CA, USA).

### 4.8. Assessment of Mitochondrial Membrane Potential

A375.S2 cells were seeded onto 96-well plates and incubated overnight. Following treatment with varying concentrations of cudraflavone C for 24 h, the cells were stained with JC-1(5,5,6,6′-Tetrachloro-1,1′,3,3′-tetraethylbenzimidazolylcarbocyanineiodide; Sigma-Aldrich). The cells were incubated at 37 °C for 30 min and analyzed using a fluorescence spectrometer at 590 nm (for red fluorescence) and 520 nm (for green fluorescence). The fluorescence ratio (590/520 nm) represented the change in mitochondrial membrane potential.

### 4.9. Determination of Mitochondrial Membrane Potential

A375.S2 cells were seeded onto 12-well plates at 37 °C and 5% CO_2_, and treated with cudraflavone C at various time intervals. Following treatment, cells were incubated with 10µg/mL JC-1 at 37 °C for 30 min and analyzed using a the BD FACScan flow cytometer (BD Biosciences, San Jose, CA, USA) at 590nm (for red fluorescence) and 520 nm (for green fluorescence). The fluorescence ratio (590/520 nm) represented the change in mitochondrial membrane potential.

### 4.10. Determination of Cellular ROS Production

Cellular ROS production was measured using the CellROX assay. A375.S2 cells were seeded onto 12-well plates and incubated overnight. Cells were treated with different concentrations of cudraflavone C (with or without inhibitors) for different time intervals, and stained with CellROXGreen Reagent (Life Technologies, Carlsbad, CA, USA). The fluorescence intensity of the cells was assessed using flow cytometry (excitation wavelength: 495 nm; emission wavelength: 529 nm) (Accuri C6 Flow Cytometer) (BD Biosciences, San Jose, CA, USA).

### 4.11. Determination of Mitochondrial ROS Production

Mitochondrial ROS production was evaluated using the MitoSOX assay. A375.S2 cells were seeded onto 12-well plates and incubated overnight. Cells were treated with different concentrations of cudraflavone C (with or without inhibitors) for different time intervals, and stained with MitoSOX Red mitochondrial superoxide indicator (Molecular Probes). The fluorescence intensity of the cells was assessed using flow cytometry (excitation wavelength: 510 nm; emission wavelength: 580 nm). 

### 4.12. Western Blotting

A375.S2 cells were seeded onto 12-well plates and incubated overnight. Cells were treated with 10 µM cudraflavone C (with or without inhibitors) for different time intervals. Following treatment, the cells were lysed in the lysis buffer and subjected to protein extraction. Equal amounts of proteins were used forperforming sodium dodecyl sulfate-polyacrylamide gel electrophoresis (SDS-PAGE) (Bio-Rad Laborator, Hercules, CA, USA). Subsequently, the proteins were transferred onto nitrocellulose membranes, which were incubated separate overnight with the primary antibodies for phospho-p38, phospho-ERK, phospho-JNK, Puma, Bax, Bad, Bid, Apaf-1, cytochrome c, caspase-3, caspase-7, and caspase-9. Next, secondary antibodies conjugated with horseradish peroxidase were added and immunoreactivity was assessed using the enhanced chemiluminescence (ECL) detection system.

### 4.13. Determination of Caspase Activities

The activities of different caspases in cell lysates were evaluated using the caspase-3, caspase-7, and caspase-9 colorimetric assay kits, as per the manufacturer’s protocol. Following treatment with cudraflavone C (with or without inhibitors), A375.S2 cells were lysed in the lysis buffer. Specific substrates for caspase-3, caspase-7, and caspase-9 (Ac-DEVD-pNA and Ac-LEHD-pNA) were added to the cell lysates (50 µg proteins) and the samples were incubated at 37 °C for 1 h. The absorbance was measured at 405 nm using a plate reader.

### 4.14. Statistical Analysis

All data were analyzed using GraphPad Prism (GraphPad, San Diego, CA, USA). Results were expressed as mean ± standard error of the mean (SEM). Continuous data between two groups were compared using the unpaired t-test. Analysis of variance (ANOVA) was used to compare continuous data between multiple groups. A *p*-value < 0.05 was considered to be statistically significant.
